# mxfda: a comprehensive toolkit for functional data analysis of single-cell spatial data

**DOI:** 10.1093/bioadv/vbae155

**Published:** 2024-11-13

**Authors:** Julia Wrobel, Alex C Soupir, Mitchell T Hayes, Lauren C Peres, Thao Vu, Andrew Leroux, Brooke L Fridley

**Affiliations:** Department of Biostatistics & Bioinformatics, Emory University, Atlanta, GA 30322, United States; Department of Biostatistics & Bioinformatics, H. Lee Moffitt Cancer Center and Research Institute, Tampa, FL 33612, United States; Department of Genitourinary Oncology, H. Lee Moffitt Cancer Center and Research Institute, Tampa, FL 33612, United States; Department of Cancer Epidemiology, H. Lee Moffitt Cancer Center and Research Institute, Tampa, FL 33612, United States; Department of Biostatistics & Informatics, Colorado School of Public Health, Aurora, CO 80045, United States; Department of Biostatistics & Informatics, Colorado School of Public Health, Aurora, CO 80045, United States; Division of Health Services & Outcomes Research, Children’s Mercy, Kansas City, MO 64108, United States

## Abstract

**Summary:**

Technologies that produce spatial single-cell (SC) data have revolutionized the study of tissue microstructures and promise to advance personalized treatment of cancer by revealing new insights about the tumor microenvironment. Functional data analysis (FDA) is an ideal analytic framework for connecting cell spatial relationships to patient outcomes, but can be challenging to implement. To address this need, we present mxfda, an R package for end-to-end analysis of SC spatial data using FDA. mxfda implements a suite of methods to facilitate spatial analysis of SC imaging data using FDA techniques.

**Availability and implementation:**

The mxfda R package is freely available at https://cran.r-project.org/package=mxfda and has detailed documentation, including four vignettes, available at http://juliawrobel.com/mxfda/.

## 1 Introduction

Advancements in single-cell (SC) spatial technologies have enabled researchers to study tissue structure and function at a cellular level while preserving the original spatial context of the tissue (Vandereyken *et al.* 2023, Wrobel *et al.* 2023, Liu *et al.* 2024). New technologies are rapidly emerging and typically fall into two categories: (1) those that measure protein abundance *in situ*, including multiplex immunofluorescence and imaging mass cytometry (Giesen *et al.* 2014, Tan *et al.* 2020), and (2) spatially-resolved transcriptomics assays that target mRNA (Ståhl *et al.* 2016). Though these types of technologies differ substantially in their preprocessing pipelines, at the downstream analysis level both promise the discovery of novel spatial relationships among different cell types and how these relationships relate to patient outcomes (Bressan *et al.* 2023). It remains a challenge to extract spatial information that fully characterizes clinically meaningful patient phenotypes from these data.

To this end, spatial summary functions from the spatial point process literature, such as Ripley’s K, can be used to quantify the clustering and co-occurrence of cells in a sample (Wilson *et al.* 2021). In this framework, the locations of cells are treated as following a point process, and realizations of a point process are called “point patterns”. Under the assumption that the rate of a cell type of interest is constant over an entire tissue, a point pattern will exhibit complete spatial randomness (CSR). The key question is if the observed pattern, as measured by a spatial summary function, deviates from CSR through clustering (Wilson *et al.* 2021). Clinically meaningful clustering patterns can then be assessed by using this spatial summary metric (computed for each tissue sample) as a covariate in a regression model of patient outcomes, such as survival or treatment response. This approach has been used to show that the degree of clustering of different types of immune cells is significantly associated with overall survival in ovarian and breast cancers (Keren *et al.* 2018, Wilson *et al.* 2022).

Several software packages for spatial analysis of SC data have recently emerged (Creed *et al.* 2021, Canete *et al.* 2022, Palla *et al.* 2022, Ehsani *et al.* 2023, Masotti *et al.* 2023, Windhager *et al.* 2023, Marconato *et al.* 2024, Samorodnitsky *et al.* 2024). However, many of these methods calculate spatial metrics at a particular predetermined distance or radius, *r*, and the selection of this distance can be arbitrary. An alternative to calculating a single spatial value at radius *r* for each sample is to perform inference using the entire spatial summary function or curve evaluated over a range of distances covering the spatial domain. Methods from functional data analysis (FDA), an area of statistics that treats entire curves as predictors or outcomes in linear models (Crainiceanu *et al.* 2024), are well suited to this task. Because functional regression models can capture highly nonlinear patterns over space or time, they have played a critical role in other areas of computational biology (Cremona *et al.* 2019, Seal *et al.* 2024). However, the adoption of FDA methods in computational biology has lagged behind other statistical or machine learning approaches, in part due to a lack of user-friendly software.

To address this gap, we introduce mxfda, an R package for FDA of SC spatial data, with custom tools for data wrangling, modeling, and visualization. Extending methodology described in Vu *et al.* (2022, 2023), we intend to set a foundation for FDA of spatial point process data from biological studies. The mxfda package has extensive documentation, including four vignettes detailing different aspects of the FDA pipeline for spatial SC data: (1) mx_fda, which explains how to set up an mxFDA S4 object and estimate spatial summary functions from the cell spatial coordinates, (2) mx_fpca, which describes dimension reduction and data exploration using functional principal component analysis (FPCA), (3) mx_funreg, which explains how to model patient outcomes using functional regression models with spatial summary functions as covariates, and (4) a vignette detailing how to convert spatial transcriptomics data to the mxFDA format. One key feature of the package is the ability to incorporate user-defined spatial summary functions, in addition to existing methods. This flexibility allows users to utilize continuous-valued information, such as transcript counts or protein expression, rather than focusing solely on cell phenotype information, which is our primary emphasis. Moreover, the vignettes are constructed using data from real spatial SC experiments of non-small cell lung carcinoma and ovarian cancer.

## 2 Package workflow

**Figure 1.**
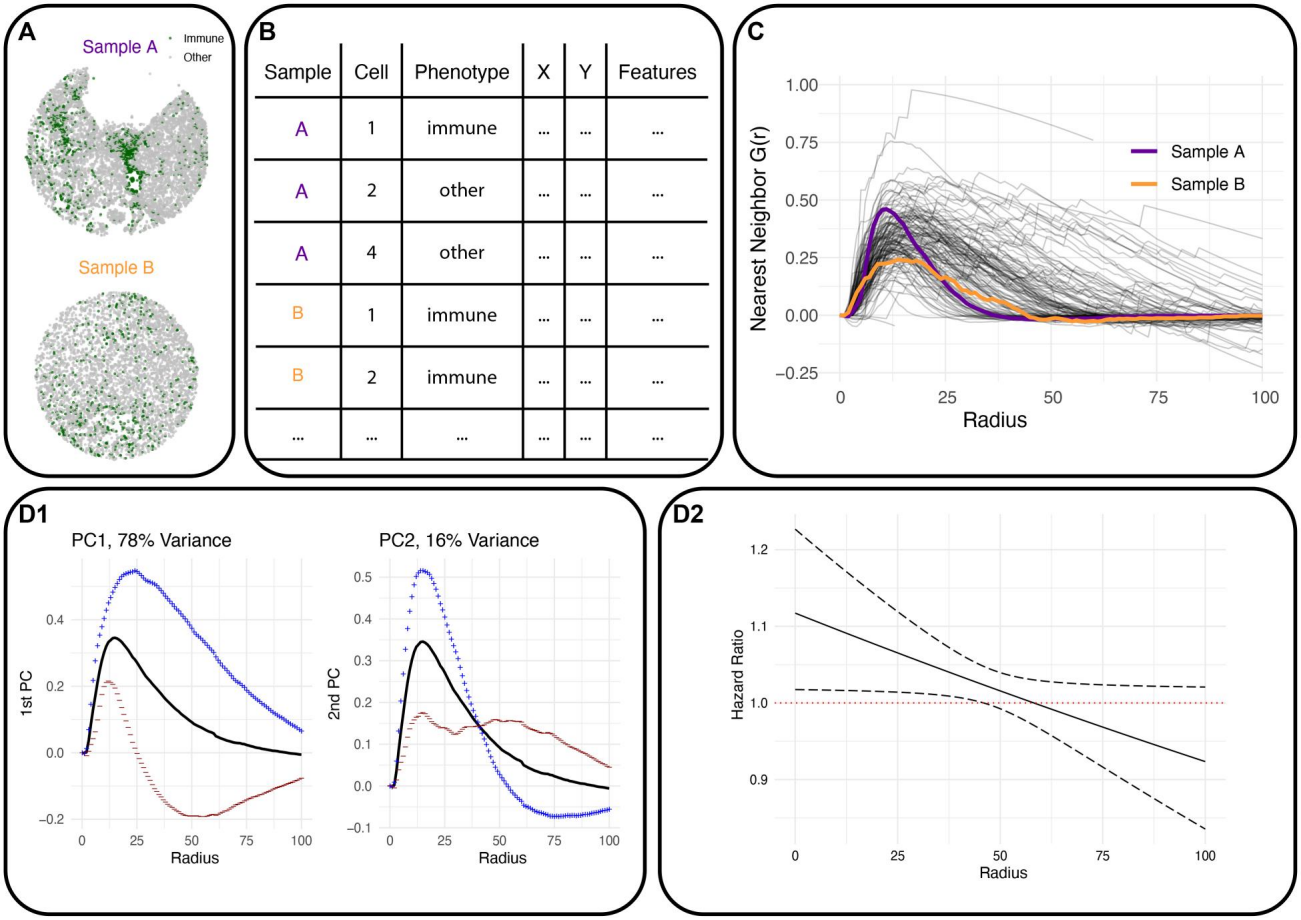
Typical workflow for the mxfda package. **A** shows the arrangement of immune (bold) and all other cells in two ovarian cancer samples. **B** depicts a typical single-cell spatial dataset, with a row for each cell containing spatial coordinates, cell phenotype, and patient features; this is transformed into an object of class “mxFDA” using the make_mxfda() function. **C** shows G(r) for each of the 128 ovarian cancer patients in the dataset, estimated using extract_summary_functions(). Highlighted are summary functions for the two samples in (A). **D1** shows the mean (solid line) ± one standard deviation (dotted lines) of the first (left panel) or second (right panel) principal component. FPCA is estimated using run_fpca() and visualized using plot(). **D2** shows the hazard ratio (solid line) and 95% confidence bands (dotted lines) from a functional Cox model, estimated using run_fcm().

In the following sections, we present a workflow for performing FDA with spatial SC data (Fig. 1). First, we describe the data structure and how to format the data for the mxfda package. Next, we show how each sample is characterized using a spatial point process. Finally, we explain methods for FPCA and functional regression implemented in the mxfda package that can be used to model the relationship between tissue spatial structure and patient outcomes.

### 2.1 Datasets

All examples in the mxfda package use data adapted from the Bioconductor package VectraPolarisData. VectraPolarisData contains data from two multiplex imaging experiments conducted at the University of Colorado Anschutz Medical Campus, one study involving 128 patients with high-grade serous ovarian cancer (Steinhart *et al.* 2021), and a second study consisting of 153 patients with non-small cell lung carcinoma (Johnson *et al.* 2021). Each dataset contains spatial coordinates and other sample characteristics for over 1.5 million cells. Code to reproduce the analysis for Fig. 1 is provided in the Supplement.

### 2.2 Configuring the mxFDA object

The mxfda package is built to work with spatial SC data that has already undergone image preprocessing steps such as cell and tissue segmentation (Blampey *et al.* 2024), batch correction (Korsunsky *et al.* 2019, Harris *et al.* 2022), and cell phenotyping (Bortolomeazzi *et al.* 2022, Xiong *et al.* 2024). After these preprocessing steps have been completed, samples are typically stored in a tabular format where each row is a cell, and each column is a feature including cell X and Y spatial coordinates, cell phenotype, and patient demographics and outcome variables (Fig. 1B). Analyses for the mxfda package are executed and stored using an S4 object of class “mxFDA”, where tabular data from Fig. 1B is converted to an “mxFDA” object using the make_mxfda() function. The “mxFDA” object provides a data structure ensuring functions from the mxfda package can expect consistent data formats, while also enabling custom behavior of common S3 methods such as summary() and plot().

### 2.3 Extracting spatial summary functions

Figure 1A shows the spatial distributions of cells from two patients from the ovarian cancer dataset, with immune and other cells labeled green and gray, respectively. Our goal is to extract a spatial summary metric from each image that summarizes the spatial clustering of immune cells as a function of radius. This spatial measure is then used as a covariate in a statistical model. Spatial methods from the geospatial statistics literature, including Ripley’s *K* and nearest neighbor *G*, have become popular for summarizing cell-type clustering in spatial SC data (Wilson *et al.* 2021). The *K* and *G* statistics have both univariate and bivariate forms and are intended to capture clustering of a single cell type or colocalization of two different cell types, respectively.

Mathematically, univariate *K* is given by

$$K(r)=\frac{\left|A\right|}{m(m-1)}\sum_{i=1}^{m}\sum_{i\neq j}^{m}1\left(d\left\{c_{i},c_{j}\right\}\leq r\right)e_{ij},$$

where $d\left\{c_{i},c_{j}\right\}$ is the pairwise distance between cells $c_{i}$ and $c_{j}$, |*A*| is the tissue area, 1(·) is an indicator function, and the $e_{ij}$ is an edge correction to account for bias that occurs for points at the boundary of the tissue region. Similarly, univariate G(r) is the probability that the nearest cell of type $c_{1}$ lies within a radius *r* of a cell of the same type, and is defined as:

$$G(r)=\frac{1}{m}\sum_{i=1}^{m}1\left(d_{NN}\left\{c_{1i}\right\}r\right)$$

where $d_{NN}\left\{c_{1}\right\}$ is the nearest-neighbor distance for cell type $c_{1}$, defined as the shortest distance between a specific point in a point pattern and its closest neighboring point. For a discussion of edge corrections for both *K* and *G* see Baddeley *et al.* (2015).

The function mxfda::extract_summary_functions() is used to estimate the spatial summary function for each sample. The user can choose between univariate, bivariate, and a multivariate metric based on entropy from Vu *et al.* (2023). The extract_summary_functions() function accepts two primary arguments: ‘extract_func’, which specifies whether to use a univariate, bivariate, or multivariate summary, and ‘summary_func’, which determines the type of summary function (such as *G* or *K*) using function names from the spatstat package. For instance, to compute the bivariate Ripley’s *K* function, the code would be structured as follows:

   extract_summary_functions(extract_func = bivariate, summary_func = Kcross).

In addition, the user can choose to compare to a theoretical version of CSR or a more robust empirical version based on permutations of cell labels to account for regions of the tissue where no cells were able to be measured (i.e., holes in the tissue) (Wilson *et al.* 2022).

The user can also supply a different spatial summary metric than the ones provided in the package; how to customize this aspect of the pipeline is described in the mx_fda vignette.

Once estimated, spatial summary functions are stored as part of the mxfda object, and can be visualized using the plot() function. Figure 1C shows nearest neighbor *G* functions, G(r), for the 128 patients in the ovarian cancer dataset. Each line represents G(r) for a specific patient and can be interpreted as the probability beyond chance of observing a neighboring immune cell within radius *r*. At a radius r<25, sample A has more clustered immune cells, and this is captured by a higher G(r) value, than sample B.

### 2.4 Functional data models

Once spatial summary functions have been extracted, the next step in the pipeline is to conduct FDA. Popular FDA methods relevant to the analysis of SC spatial data include FPCA and functional regression, and implementations of both are included in mxfda. We describe these methods briefly here, but refer interested readers to (Crainiceanu *et al.* 2024) for a recently published overview of common FDA methods. FPCA, the analog of principal components (PCs) analysis for functional data, characterizes dominant patterns in the data and is frequently used for dimension reduction and clustering. Functional regression is the FDA analog of (generalized) linear regression, where a function can be the outcome, a predictor, or both. In the context of SC data, these regression models allow users to perform estimation and inference on the association between patient outcomes and spatial clustering simultaneously for all radii *r*.

FPCA is estimated using the run_fpca() function and visualized using plot() (Xiao *et al.* 2016). Figure 1D1 shows the results of running FPCA on the G(r) curves presented in Fig. 1C. The black line represents the mean curve, while the blue and red dotted lines show ± one standard deviation of the first (left panel) or second (right panel) PC. The mean curve shows that, on average across all samples, *G* is highest at approximately r=15 and decreases as *r* increases. The first PC, which explains 78% of the variance in the curves, can be interpreted as a shift up or down from the population mean. The second PC, which explains 16% of the variance, reflects either a pattern of more clustering than average at r<50 but less clustering at r>50 (blue line) or a relatively consistent *G* across radii (red line). Each individual’s *G* curve is a linear combination of the patterns represented by each PC. For instance, a subject with a high score for FPC1 and scores near zero for other PCs will have a curve that closely resembles the blue line for PC1. Multilevel FPCA (MFPCA), a method for dimension-reduction when there are multiple samples per patient, is also implemented in mxfda. Further examples with FPCA and MFPCA are provided in the package vignette mx_fpca.

The mxfda package also implements scalar-on-function regression, in which the outcome is a scalar patient characteristic such as survival or disease subtype, and spatial summary functions from Fig. 1C are the modeled as covariates. Specifically, mxfda allows for models with survival outcomes described in Cui *et al.* (2021); Vu *et al.* (2022) using the run_fcm() function, and binary and continuous outcomes using the run_sofr() function. In these regression models, the association between spatial summary functions, denoted X(r), and the outcome, denoted *Y*, is estimated through a *functional regression coefficient*, β(r). β(r) is interpreted the same as a standard regression coefficient, with the addition that it may have a different value at each radius *r*. Figure 1D2 shows $e^{\beta (r)}$ presents results from a functional Cox regression model to determine the impact of the curves in Fig. 1C on overall survival in ovarian cancer. In survival models the exponentiated coefficient is interpreted as a hazard ratio (HR), given by the solid black line. The dotted black lines show the 95% confidence interval at each radius *r*, and regions where the dotted black lines do not contain the horizontal red line (i.e., HR = 1) are statistically significant. Figure 1D2 indicates that greater immune cell clustering for r∈(0,45) is significantly associated with better survival. Plots of functional regression coefficients can be quickly made using mxfda::plot(). More functional regression details are available in the mx_funreg vignette.

## 3 Conclusion

The tools provided in the mxfda package enable biomedical researchers to implement a wide range of FDA methods for spatial SC data, and perform inference on the relationship between spatial clustering of different cell types and patient outcomes at a range of distances covering the sample spatial domain. All vignettes and package functions are illustrated using examples with open-source data from real SC experiments to demonstrate how researchers can apply these methods to their own data. Taken together, the mxfda package facilitates a unique approach to the spatial analysis of SC transcriptomics and proteomics data with potential for broad application in biomedical research.
